# Paucity of Intact Non-Induced Provirus with Early, Long-Term Antiretroviral Therapy of Perinatal HIV Infection

**DOI:** 10.1371/journal.pone.0170548

**Published:** 2017-02-08

**Authors:** Kaitlin Rainwater-Lovett, Carrie Ziemniak, Douglas Watson, Katherine Luzuriaga, George Siberry, Ann Petru, YaHui Chen, Priyanka Uprety, Margaret McManus, Ya-Chi Ho, Susanna L. Lamers, Deborah Persaud

**Affiliations:** 1 Department of Pediatrics-Infectious Diseases, Johns Hopkins University School of Medicine, Baltimore, MD, United States of America; 2 Department of Pediatrics, University of Maryland, Baltimore, MD, United States of America; 3 Program in Molecular Medicine, University of Massachusetts Medical School, Worcester, MA, United States of America; 4 Maternal and Pediatric Infectious Disease Branch, Eunice Kennedy Shriver National Institute of Child Health and Human Development, Rockville, MD, United States of America; 5 Department of Pediatric Infectious Diseases, Children's Hospital and Research Center Oakland, Oakland, CA, United States of America; 6 Department of Medicine-Infectious Diseases, Johns Hopkins University School of Medicine, Baltimore, MD, United States of America; 7 Bioinfoexperts LLC, Thibodaux, LA, United States of America; University of Pittsburgh Centre for Vaccine Research, UNITED STATES

## Abstract

The latent reservoir is a major barrier to HIV eradication. Reservoir size is emerging as an important biomarker to assess the likelihood of HIV remission in the absence of antiretroviral therapy (ART) and may be reduced by earlier initiation of ART that restricts HIV spread into CD4+ T cells. Reservoir size is traditionally measured with a quantitative viral outgrowth assay (QVOA) that induces replication-competent HIV production through *in vitro* stimulation of resting CD4+ T cells. However, the recent identification of replication-intact, non-induced proviral genomes (NIPG) suggests the QVOA significantly underestimates (by 62-fold) latent reservoir size in chronically-infected adults. Whether formation and persistence of Intact, NIPG is thwarted by early ART initiation and long-term virologic suppression in perinatal infection is unclear. Here, we show that the latent reservoir in 11 early treated, long-term suppressed perinatally infected children and adolescents was not inducible by QVOA and dominated by defective, NIPG. Single genome analysis of 164 NIPG from 232 million cultured resting CD4+ T cells revealed no replication-intact, near-full length sequences. Forty-three (26%) NIPG contained APOBEC3G-mediated hypermutation, 115 (70%) NIPG contained large internal deletions, one NIPG contained nonsense mutations and indels, and 5 (3%) NIPG were assigned as “Not Evaluable” due to multiple failed sequencing attempts that precluded further classification. The lack of replication competent inducible provirus and intact NIPG in this cohort indicate early, long-term ART of perinatal infection leads to marked diminution of replication-competent HIV-1 reservoirs, creating a favorable state towards interventions aimed at virologic remission.

## Introduction

Infection with HIV results in the rapid formation of a latent reservoir in resting memory CD4+ T cells (rCD4s) that cannot be eradicated by combination antiretroviral therapy (ART) and is capable of rekindling viremia after ART discontinuation [1–5]. The size of the rCD4 latent reservoir has been associated with the risk and timing of virologic rebound following ART cessation [6–9]. Traditionally, the presence of inducible latent provirus is detected by a quantitative viral outgrowth assay (QVOA) in which a single-round of CD4+ T cell activation is used to reverse HIV latency and induce infectious virus production from rCD4s to infect susceptible target cells, allowing quantitative estimates of latent reservoir size [10]. However, replication-competent, non-induced proviral genomes (NIPG) were recently detected in culture- negative wells of the standard QVOA indicating that this measure underestimates the size of the replication-competent latent reservoir by up to 62-fold in chronically-infected adults [11].

Earlier antiretroviral therapy (ART) initiation in perinatal infection was associated with smaller reservoir size in later childhood, as measured by the QVOA [12]. Studies in adults have also shown smaller reservoir sizes with early ART [13–15]. Other molecular biomarkers of HIV persistence, such as cell-associated HIV DNA and RNA concentrations, also indicate reservoir diminution over time following early, long-term virologic suppression, whether through loss of HIV-infected cells or dilution by new, uninfected cell populations [12,16–20], but these methods detect both replication-competent and -defective genomes. In a recent study of HIV-infected adults, treatment with ART during acute infection led to a lower proportion of intact HIV proviruses relative to defective HIV proviruses in rCD4s [21], supporting early and rapid accumulation of defective genomes such as those generated from innate immune mechanisms like APOBEC3G [22]. Given earlier initiation of long-term, suppressive ART and fundamental differences in immunity relative to adults, we expected early ART initiation in perinatal infection to result in a higher proportion of intact NIPG relative to defective genomes. Effective ART likely arrests reservoir seeding, as indicated by smaller reservoir size with earlier ART initiation (reviewed in [23]). Additionally, ART initiation during infancy curtails the development of HIV-specific immunity (reviewed in [18]), restricting genome alterations. To evaluate this hypothesis, we used a previously described, rigorous method [11,21] to identify intact NIPG in the standard QVOA using near full-length HIV-1 proviral sequencing on peripheral blood mononuclear cells enriched for resting CD4+ T cells from perinatally-infected children and adolescents who have maintained virologic suppression since initiating ART before six months of age.

## Results

### Screening for NIPG

**Fig 1:**
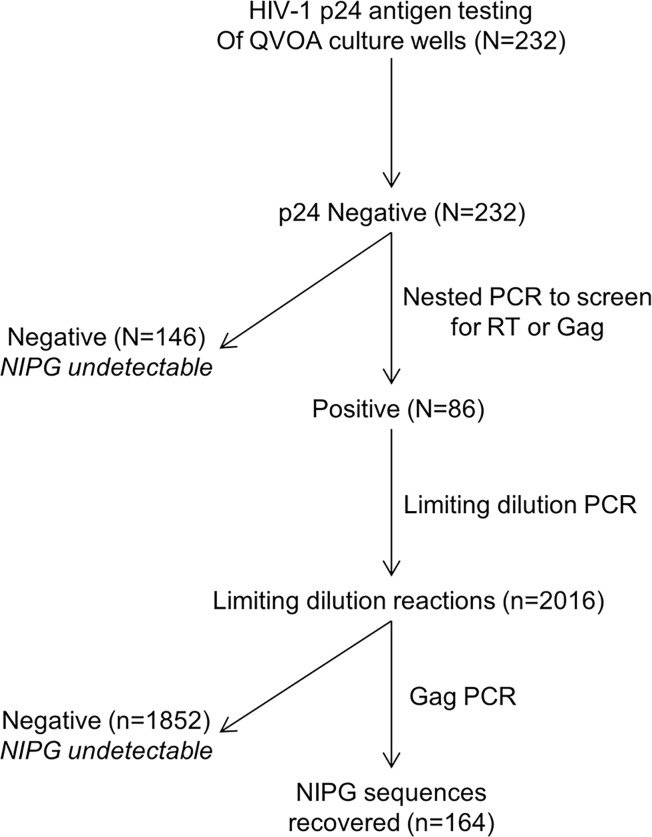
Flow diagram of QVOA wells yielding NIPG. “N” represents the number of QVOA culture wells; “n” represents the number of PCR reactions performed at limiting dilution and therefore, the number of NIPG recovered at a clonal level. *QVOA*: *quantitative viral outgrowth assay*, *NIPG*: *non-induced proviral genomes*, *PCR*: *polymerase chain reaction*.

To determine the extent to which early, long-term treatment influences the composition of the latent reservoir, the presence of induced replication-competent genomes was quantified and characterized using the QVOA. A total of 232-culture wells (median: 16 wells per study participant) each containing one million rCD4s were assayed from 11 perinatally-infected children and adolescents who initiated ART at a median of 8.9 weeks of age (IQR: 7.9, 10.0), suppressed viremia at a median of 4.2 months of age (IQR: 3.9, 4.5), and continued ART without interruption or rebound viremia for a median of 11.0 years (IQR: 10.4, 14.8) (Table 1; S1 Fig). The median age at time of study was 11.1 years (IQR: 10.6, 15.0) (Table 1). Inducible, replication-competent HIV was not detected by p24 antigen production in the culture supernatant of any of the 232 QVOA wells from the 11 study participants (Fig 1). We screened these 232 p24-negative QVOA wells by PCR for the Gag or RT gene to identify QVOA wells that contained HIV DNA in sufficient concentrations for the near full- length genome analysis. Eighty-six wells (37%) screened positive, from which we performed 2,016 limiting dilution PCR reactions to quantify the proportion of intact NIPG.

**Table 1:** Characteristics of the study population.

Study Participant^a^	Age at ART Initiation (weeks)	Age at virologic suppression (months)	Current Age (years)	Duration of ART (years)	# resting CD4+ T cells analyzed (x10^6^)	Inducible HIV by QVOA	Western Blot Interpretation	# NIPG
A	6.7	4.5	13.5	13.4	30	No	Neg	50
C	7.1	3.5	15.3	15.1	15	No	Neg	3
E.4	9.6	4.0	10.6	10.4	28	No	*na*	20
E.6	9.6	4.0	10.8	10.6	16	No	*na*	12
E.7	9.6	4.0	11.1	10.9	12	No	+/-	19
G	17.1	5.6	7.0	6.6	13	No	+/-	2
I	11.4	4.4	17.7	17.5	24	No	Neg	4
M	10.3	4.2	4.3	4.1	9	No	+/-	7
N	8.6	4.8	15.0	14.8	20	No	Neg	44
O	8.9	3.7	6.6	6.5	25	No	+/-	3
D	9.7	4.4	11.1	11.0	6	No	+/-	0
R	6.1	3.3	15.7	15.6	12	No	Neg	0
S	8.6	4.0	12.5	12.4	22	No	+/-	0
**Median**	**8.9 weeks**	**4.2 months**	**11.1 years**	**11.0 years**	**16 x10**^**6**^ **cells**	**--**	**--**	**9.5 NIPG**^b^
**(IQR)**	**(7.9, 10.0)**	**(3.9, 4.5)**	**(10.6, 15.0)**	**(10.4, 14.8)**	**(12, 24)**			**(3.3, 19.8)**

Abbreviations: QVOA, quantitative viral outgrowth assay; NIPG, non-induced proviral genome; Neg, negative; +/-, indeterminate; na, not available.

^a^*Letter indicates participant; number indicates study visit if multiple visits.*

^b^*Median (IQR) among the eight participants with NIPG recovered.*

### Gag Sequence Analysis

**Fig 2:**
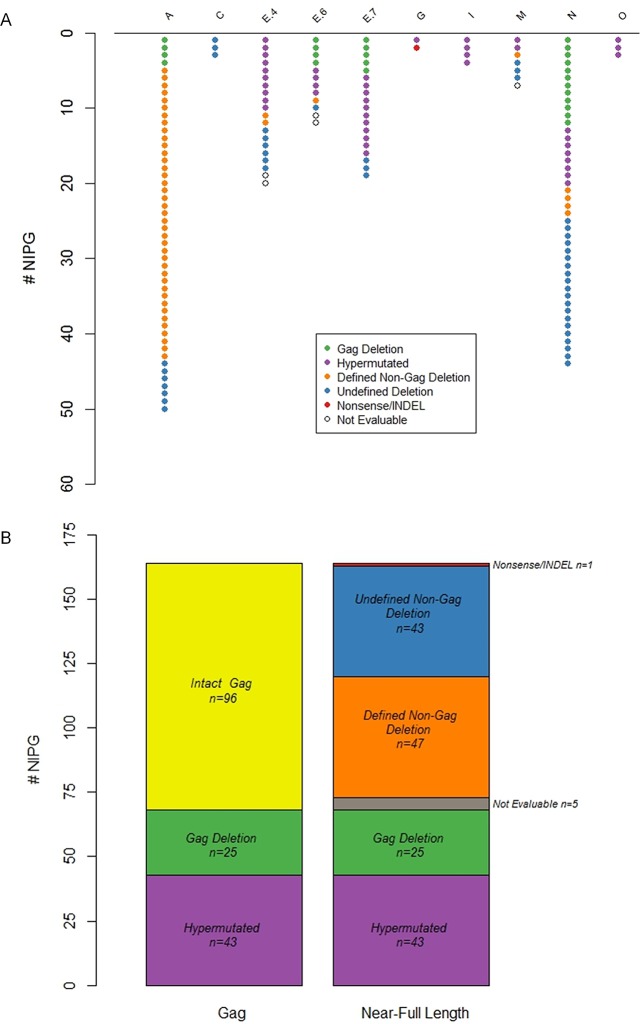
Classification of NIPG. (A) Distribution of NIPG within each study participant. Purple: hypermutation; Green: Gag deletion; Red: intact near-full length, replication-deficient due to nonsense mutations or INDELs; Blue: undefined non-Gag deletion; Orange: defined non-Gag deletion; empty circle: not evaluable. (B) Distribution of NIPG across study participants after Gag and near-full length sequencing. *NIPG*: *non-induced proviral genomes*.

One-hundred sixty-four NIPG Gag sequences were recovered from 50 of the 86 cultured wells that screened positive for either Gag or RT from eight of the 11 study participants (Fig 2A). The number of NIPG was not correlated with the number of cultured rCD4s (rho = 0.34; p = 0.282 by Spearman’s rank). Ninety-six (59%) NIPG Gag sequences were intact, 43 (26%) contained APOBEC3G-mediated G-to-A hypermutations, and 25 (15%) contained deletions within the Gag gene (Fig 2B).

### Full-Length Sequence Analysis

We found that early, long-term virologic suppression of perinatal HIV infection resulted in the persistence of defective NIPG. There also was no systematic alteration in the generation of replication-defective genomes, as evidenced by various patterns of NIPG classification between participants (Fig 2A). Near-full length sequencing of the 96 NIPG with intact Gag genes yielded an additional 90 deletions, resulting in a total of 115 deletions among the 164 NIPG (70%) (Fig 2B). Forty-three NIPG contained deletions with undefined junctions. We were unable to amplify additional genomic regions of 5 NIPG (“Not Evaluable” in Fig 2B). No additional hypermutations were identified after near-full length sequencing, yielding a total of 43 (25%) hypermutated NIPG.

**Fig 3:**
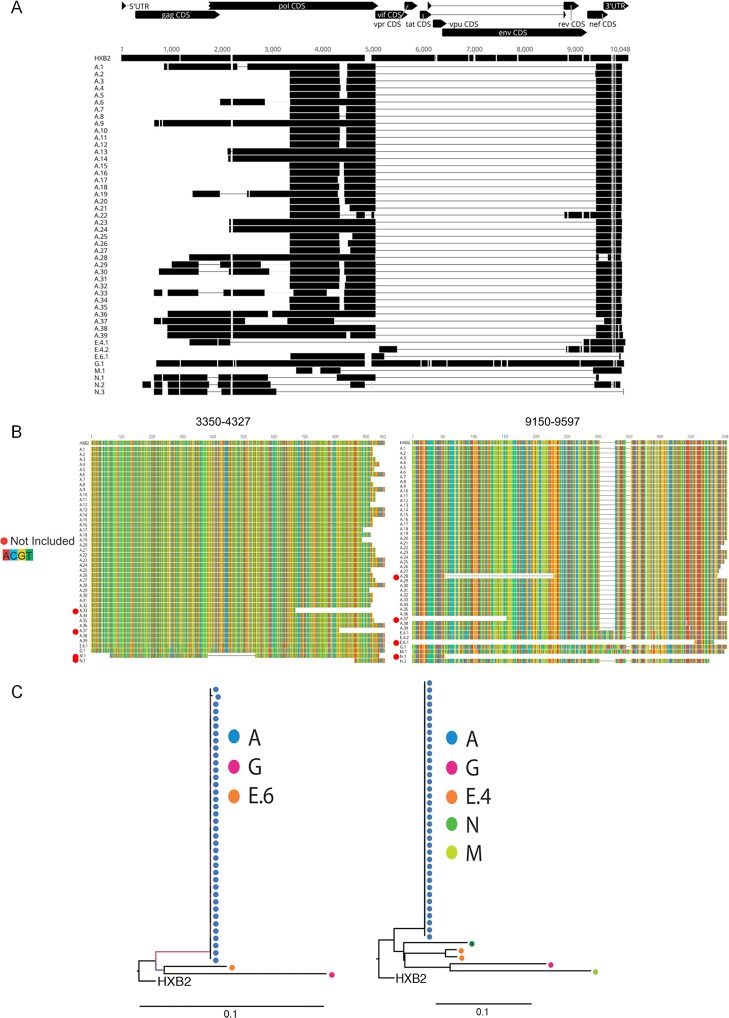
Deletions in NIPG with defined junctions. Letters and numbers correspond to participant identifiers listed in Table 1. A) Sequences are aligned to HXB2. Sequenced positions appear as a black polygon. Gray thin lines indicate deleted regions of genomes. B) Regions with relatively high coverage were extracted for phylogenetic analysis. Location shown is according to HXB2 numbering. Colors correspond to nucleotides on the left. Sequences designated with a red dot were not used in subsequent analysis due to missing sequence segments. C) Phylogenetic analysis of sequences rooted using HXB2 and aligned to positions 3350–4327 and 9105–9597.

We identified defined deletion junctions for 46 NIPG among five study participants, 39 NIPG were from study participant A (Fig 3A). Thirty of 39 sequences from study participant A contained a deletion in integrase (HXB2 ~4290–4480); however, the deletions were rarely identical because they started or stopped at slightly different positions. Additionally, 23 of 39 sequences from study participant A were completely missing gag and started in the middle of RT (HXB2 ~3300). Interestingly, A.13, A.14, A.23, and A.24 contained only the pol and LTR and all of these sequences were missing 9–47 nucleotides at the start of pol. Most (41/46) sequences had an intact 3' LTR. Sequences A.28 and A.37 both had non-identical internal LTR deletions and three other sequences (N.3, N.2, E.6.1) were almost completely missing the LTR. While sequences E.6.1 and A.22 contained nef and E.4.1 contained a partial nef, all other sequences with deletions were missing nef (other than the portion that overlaps the LTR), env and accessory genes. Only one NIPG had a near-full length genome but contained nonsense mutations and insertions/deletions (“Nonsense/Indel” in Fig 2B; G.1 in Fig 3A).

Minimal, if any, diversity was identified among nucleic acids within a study participant's sequence population (Fig 3B). Study participant E had three highly diverse sequences among the regions sequenced. Sequences A.10 and A.11 were completely identical, as were A.23 and A.24, suggesting the possibility that at least some sequences were derived from clonally expanded cells. While all NIPGs contained partial pol sequence (E.4.1 contained a very small portion), diversity mainly occurred due to varied patterns of deletions (Fig 3C).

### HIV DNA Concentrations in Culture Wells

**Fig 4:**
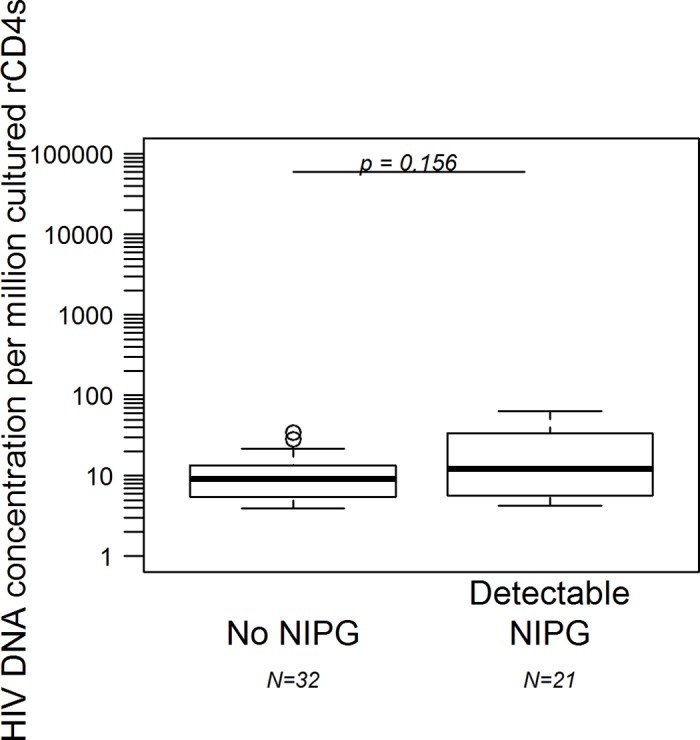
HIV DNA concentrations from cultured resting CD4+ T cell QVOA wells with and without NIPG. “N” represents the number of QVOA culture wells.

Using ddPCR, we measured the HIV DNA concentrations of a subset of cultured cells to further confirm a paucity of HIV-infected cells contributing to the low proportion of culture wells with amplifiable NIPG sequencing. HIV DNA concentrations were measured in 33 (67%) and 97 (53%) culture wells from a total of 49 p24-negative wells with detectable NIPG and 183 p24-negative wells without NIPG, respectively. Of the 97 p24-negative wells without NIPG, 65 (67%) had HIV DNA concentrations below the assay’s LOD (median LOD: 3.1 [IQR: 2.7, 3.5]). The remaining 32 p24-negative wells without NIPG had a median proviral DNA concentration of 3.1 copies per million (c/mil) cultured cells (IQR: 2.7, 3.5). Among the 33 p24-negative culture wells containing NIPG, 12 (36%) were below the assay’s LOD (median LOD: 3.0 [IQR: 2.8, 3.6]). We observed a median HIV DNA concentration of 12.2 c/million cultured cells (IQR: 5.7, 33.6) among the 21 wells above the LOD with detectable NIPG (Fig 4).

### HIV-Specific Antibody Responses

A characteristic of early treated perinatal infection is a lack of or restriction in HIV-specific antibody responses [12,16,24,25], which may reflect insufficient antigenic exposure to elicit and maintain HIV-specific antibody responses following early treatment. Previous studies report 36%-100% of early, long-term treated perinatally-infected children and adolescents as seronegative by HIV ELISA [12,16,19,26], yet further Western blot antibody testing typically shows low-level reactivity to specific HIV proteins [12,16]. Among the 11 children and adolescents in the current study with plasma available for Western blot antibody testing, none were HIV seropositive by Western blot testing. Six participants (55%) were classified as indeterminate due to weak reactivity to few HIV proteins and five (45%) were seronegative (Table 1). Few differences in Western blot profiles were observed between those with or without detectable NIPG (Table 2). One (13%) of eight participants with NIPG and one (33%) of three participants without NIPG were seropositive to the p24 protein, the most common Western blot protein to show seroreactivity (Table 2).

**Table 2:** HIV-specific antibody responses of plasma samples.

Study Participant^a^	Western Blot Interpretation	Protein-Specific Antibody Reactivity								
		gp160	gp120	p66	p55	p51	p41	p31	p24	p17
A	Neg	Neg	Neg	Neg	Neg	Neg	Neg	Neg	Neg	Neg
C	Neg	Neg	Neg	Neg	Neg	Neg	Neg	Neg	Neg	Neg
E.7^b^	+/-	+/-	Neg	+/-	Neg	Neg	+/-	Neg	**Pos**	Neg
G	+/-	Neg	Neg	Neg	Neg	Neg	Neg	Neg	+/-	+/-
I	Neg	Neg	Neg	Neg	Neg	Neg	Neg	Neg	Neg	Neg
M	+/-	Neg	Neg	+/-	Neg	+/-	Neg	Neg	+/-	Neg
N	Neg	Neg	Neg	Neg	Neg	Neg	Neg	Neg	Neg	Neg
O	+/-	Neg	Neg	Neg	Neg	Neg	Neg	Neg	Neg	+/-
D	+/-	+/-	Neg	Neg	Neg	Neg	+/-	Neg	**Pos**	+/-
R	Neg	Neg	Neg	Neg	Neg	Neg	Neg	Neg	Neg	Neg
S	+/-	Neg	Neg	Neg	Neg	Neg	Neg	Neg	+/-	Neg

Abbreviations: Neg: negative; Pos: positive; +/-: indeterminate.

^a^*Letter indicates participant; number indicates study visit if multiple visits.*

^b^*Plasma unavailable for testing from Participant E visits 4 and 6.*

## Discussion

We performed the first assessment of induced and non-induced replication-competent latent proviral genomes during early, long-term treatment to understand the distribution of replication-competent and non-induced, intact proviral genomes retained in perinatal HIV infection. Contrary to our hypothesis, HIV-infected rCD4s during early, long-term ART were dominated by replication-defective, non-intact NIPG. This profile is distinct from 12% of intact NIPG identified in chronically-infected adults, with virologic suppression [11,21], highlighting differences in HIV persistence between early and late ART initiation, but closer to the 2% recently identified with early ART in adults. A number of mechanisms governing genomic variation contribute to the generation of defective genomes during the course of HIV infection. By virtue of being a diploid virus, genome switching by reverse transcriptase results in large deletions that prove lethal [27–31]. In addition to a lack of proofreading activity that increases the rates of point mutation in HIV genomes, several innate immune factors contribute to mutagenesis, such as the cytidine deaminases APCOBEC3F and APOBEC3G that induces hypermutation [22]. Our findings confirm that early ART initiation in the context of an infant immune system with long-term virologic suppression did not lead to preservation of intact HIV genomes but persistence of an overwhelming majority of replication-defective genomes.

The extensive analysis of the NIPG proviral reservoir derived from the culture-negative wells in the QVOA conducted in this study confirmed the existence of an extremely small replication-competent reservoir after early, long-term ART in perinatal infection. A limitation of this study was the inability to distinguish between the effects of early ART initiation and long-term virologic suppression. Our previous studies showed that ART initiation by three to six months of age does not prevent establishment of the inducible latent HIV reservoir [12,16,32]. However, long-term virologic suppression may have created an environment in which natural reactivation of latently infected rCD4s promoted clearance of replication-competent genomes due to natural cellular turnover, while viral replication and seeding of the reservoir was abated with ART. This is consistent with early reservoir establishment, decreasing reservoir size with long-term effective ART [12,16,19,33–35], and the rarity of replication-competent NIPG observed in the present study.

The large number of NIPG recovered in the present study enabled us to analyze diverse sequences that yielded replication-deficient genomes. Given the relatively small number of rCD4+ T cells available for the QVOA from some adolescents, we acknowledge that this method did not fully sample the entire repertoire of proviral genomes within an individual, particularly when considering potential HIV reservoirs in non-circulating tissues such as the central nervous system and gastrointestinal tract (reviewed in [36]). While it is possible that the method was insufficiently sensitive to amplify full-length sequences after detection of the Gag gene from very low concentrations of HIV DNA in p24-negative culture wells, it is also possible that deletions or mutations existed in the region spanning the primer binding sites of the four overlapping fragments for full-length sequencing. We tested the latter by attempting to amplify shorter areas of the genome and did not recover additional sequences. We are confident the Gag products amplified were patient-specific due to the high homology within study participants between Gag sequences as illustrated in Fig 2D. Importantly, replication-competent genomes likely exist that are capable of rekindling viremia if ART is discontinued, as exemplified by the case of the Mississippi child [37,38].

Although the concentrations of HIV DNA observed here may be underestimated due to the presence of donor target cells in the QVOA, they substantiate the lack of recovery of replication-competent virus from this cohort and confirm the low concentrations observed in other studies of early treatment of adult and perinatal HIV infection [13–15,17,21,39]. Thus, measuring proviral DNA to quantify reservoir size in this population will largely reflect replication-deficient genome concentrations.

HIV antibody response profiling may also provide information on the state of viral quiescence during therapeutic interventions. Consistent with restricted or absent HIV-specific antibody responses in perinatally-infected children who receive early ART [12,16,24,25], no participants in the current study were seropositive by Western blot and only two participants were seropositive to a single Western blot protein. Seroreactivity to the Gag protein, p24, is interesting because this is one of the first HIV proteins to induce HIV-specific antibodies (reviewed in [40]). Additionally, recent reports identified that Gag can be expressed by latently infected cells without viral spread [41] and defective proviruses can encode chimeric proteins [42]. Nevertheless, the high proportion of seronegative participants in the current study and replication-defective NIPG suggests ongoing cellular expression of HIV proteins is not occurring at levels sufficient for antibody induction in this cohort. Moreover, the maintenance of absent or restricted antibody responses provides support for the high proportion of inactive viral genomes observed in this population.

Our study confirms an overwhelming dominance of replication-deficient NIPG in early, long-term treated HIV infection, evidenced by the lack of recovery of replication-competent viral clones in the QVOA and absence of detection of replication-intact NIPGs. If confirmed by other pediatric studies, the paucity of replication-competent, intact NIPG observed here differentiates the reservoir composition from that of HIV-infected adults and strengthens evidence for small reservoir size following early, long-term ART. Small blood volumes limited our ability to characterize NIPG in HIV-infected infants and children, necessitating development of new methods to advance pediatric cure research. Our and others’ [11,21] findings highlight the importance of incorporating NIPG into definitions of reservoirs and latency. Further research is needed to understand a potential shift towards defective NIPG with early, long-term ART and the clinical relevance of these genomes to serve as templates for HIV RNA transcription, protein production, and HIV-specific immune responses.

## Materials and Methods

### Study Participants

Eleven perinatally HIV-infected children and adolescents who were virologically suppressed before six months of age and remained on suppressive ART for at least four years were enrolled between April 2012 and September 2014 in an observational cohort to study HIV persistence and immune responses in perinatal infection. Youth were enrolled at four sites in the United States: Johns Hopkins University, Baltimore, MD; University of Massachusetts Medical Center, Worcester, MA; University of Maryland Institute of Human Virology, Baltimore, MD; and Children’s Hospital and Research Center, Oakland, CA. HIV antibody testing was performed at the University of Massachusetts and the remaining research was conducted at Johns Hopkins University School of Medicine.

### Ethics Statement

This study was approved by the Johns Hopkins Medicine Office of Human Subjects Research Institutional Review Boards as study number NA_00087629/CR00002960, the University of Massachusetts Medical Center Committee for the Protection of Human Subjects in Research as study dockets H00001811 and H-1327, and the Institutional Review Board at the University of Maryland as protocol number HP-00055374.

Adult study participants provided written informed consent and parental guardians of study participants younger than 18 years provided written informed consent on the child's behalf. Study participants older than 12 years provided oral assent. Study nurses from the University of Massachusetts obtained oral consent from parental guardians on behalf of two study participants who were under 18 years of age at the Children's Hospital and Research Center, Oakland. Oral consent was approved by the University of Massachusetts Medical Center Committee for the Protection of Human Subjects in Research. Parental guardians then provided written informed consent on behalf of the study participants following the enrollment discussion with study nurses.

### Quantitative Viral Outgrowth Assay

Whole blood was collected from study participants and peripheral blood mononuclear cells were isolated using Ficoll Hypaque. Resting CD4+ T cells were enriched for using bead depletion and cultured in one-million cell replicates in five-fold serial dilutions as previously described [43]. Expression of replication- competent HIV was induced through phytohemagglutinin stimulation and cells and supernatant were harvested at culture day 14 after feeding twice with PHA-stimulated, CD8-depleted PBMCs from an uninfected donor. Detection of HIV p24 antigen by enzyme-linked immunoassay (EIA) (Perkin Elmer, Waltham, MA) was used to identify replication-competent HIV from cell culture supernatant.

### Sequencing of NIPG

Total cellular DNA was isolated from each p24-negative culture well using a QIAamp DNA midi kit (Qiagen) and screened for the presence of HIV provirus by nested PCR using primers specific to the Gag or RT genes (Integrated DNA Technologies). A 9kb, near-full length product was amplified in limiting dilution format from wells screening positive for non-induced provirus, as previously described [11,21]. The Gag gene was amplified from the near-full length amplicon, bead purified (Agencourt AMPure XP), and sequenced by Sanger methods on the Applied Biosystems 3730xl DNA Analyzer in the Genetic Resources Core Facility (Johns Hopkins School of Medicine, Baltimore, MD). Gag sequences were aligned to the HXB-2 reference sequence (CodonCode Aligner v4.04) and inspected for deletions. APOBEC3G-mediated G-to-A hypermutation was assessed using the Los Alamos National Laboratory Hypermut Tool (http://www.hiv.lanl.gov/content/sequence/HYPERMUT/hypermut.html).

Four overlapping, nested PCR reactions were prepared from the 9kB, near-full length amplicons that demonstrated intact Gag sequences and purified, sequenced, and analyzed as above. Sequences were categorized as “Not Evaluable” when the 9kb template was depleted prior to obtaining near-full length sequence. Deletions with undefined junctions were defined by the identification of human sequence interspersed among HIV sequence or failure of at least three sequencing primers and PCR amplicons with smaller than expected size upon gel electrophoresis. The sensitivity of these methods was evaluated by amplification and sequencing of serial dilutions of the HIV-1 strain, BaL (data not shown). Additionally, full-length replication-competent sequences were detected from p24+ QVOA culture wells (data not shown).

Sequences were aligned to the HXB2 reference genome using the MAFTT algorithm in Geneious software (v9.1). The initial alignment was proofed and edited by hand to ensure proper placement of nucleotides in gapped/truncated columns. A neighbor-joining tree was produced for specific regions of genome with high coverage in MEGA (6.0) using the Tajima-Nei molecular model, 1000 bootstrap replicate samples for statistical support, and rooted using the HXB-2 reference sequence. Trees were viewed and colored in Figtree software v1.4 (http://tree.bio.ed.ac.uk/software/figtree).

### Droplet Digital PCR

Total HIV DNA concentrations were quantified by digital droplet PCR (ddPCR; BioRad) as previously described [44]. Concentrations below the lower limit of detection (LOD) were set to the LOD for analysis.

### HIV-Specific Antibody

Plasma was isolated from whole blood using centrifugation. HIV-specific antibodies were detected using the Cambridge Biotech HIV Western blot kit (Maxim Biomedical), as previously described [16]. Indeterminate reactivity against each Western blot protein was classified as seronegative for analytic purposes.

### Statistical Analyses

Fisher’s exact test and the Mann-Whitney U-test were used to compare dichotomous and continuous data, respectively. Correlations were evaluated using the Spearman method. Continuous variables were expressed as the median and IQR unless otherwise specified. All analyses were performed in R v3.1.0 (http://www.R-project.org) and used the ‘lattice’ package [45].

## Supporting Information

S1 Fig Plasma viral load trajectories since ART initiation. Letter above each plot corresponds to participant identifier listed in Table 1. Black dashed horizontal line indicates 400 c/mL. Red vertical line indicates time of observation for study visit/s in this report. Data preceding zero on the x-axis indicate pre-ART plasma viral load measurements. Viral loads at the limits of detection (*e*.*g*., <400, <50, etc.) were set to the limit of detection (*e*.*g*., 400, 50, etc., respectively). *ART*: *antiretroviral therapy*, *c/mL*: *copies of HIV RNA per milliliter of blood plasma*.
(PDF)
