# Supplementary figures and images for: Paucity of Intact Non-Induced Provirus with Early, Long-Term Antiretroviral Therapy of Perinatal HIV Infection
Source: PLoS One. 2017 Feb 8;12(2):e0170548. doi: 10.1371/journal.pone.0170548 (PMC5298215; doi:10.1371/journal.pone.0170548)

# Plasma viral loads over time

Plasma viral load (log<sub>10</sub> c/mL)

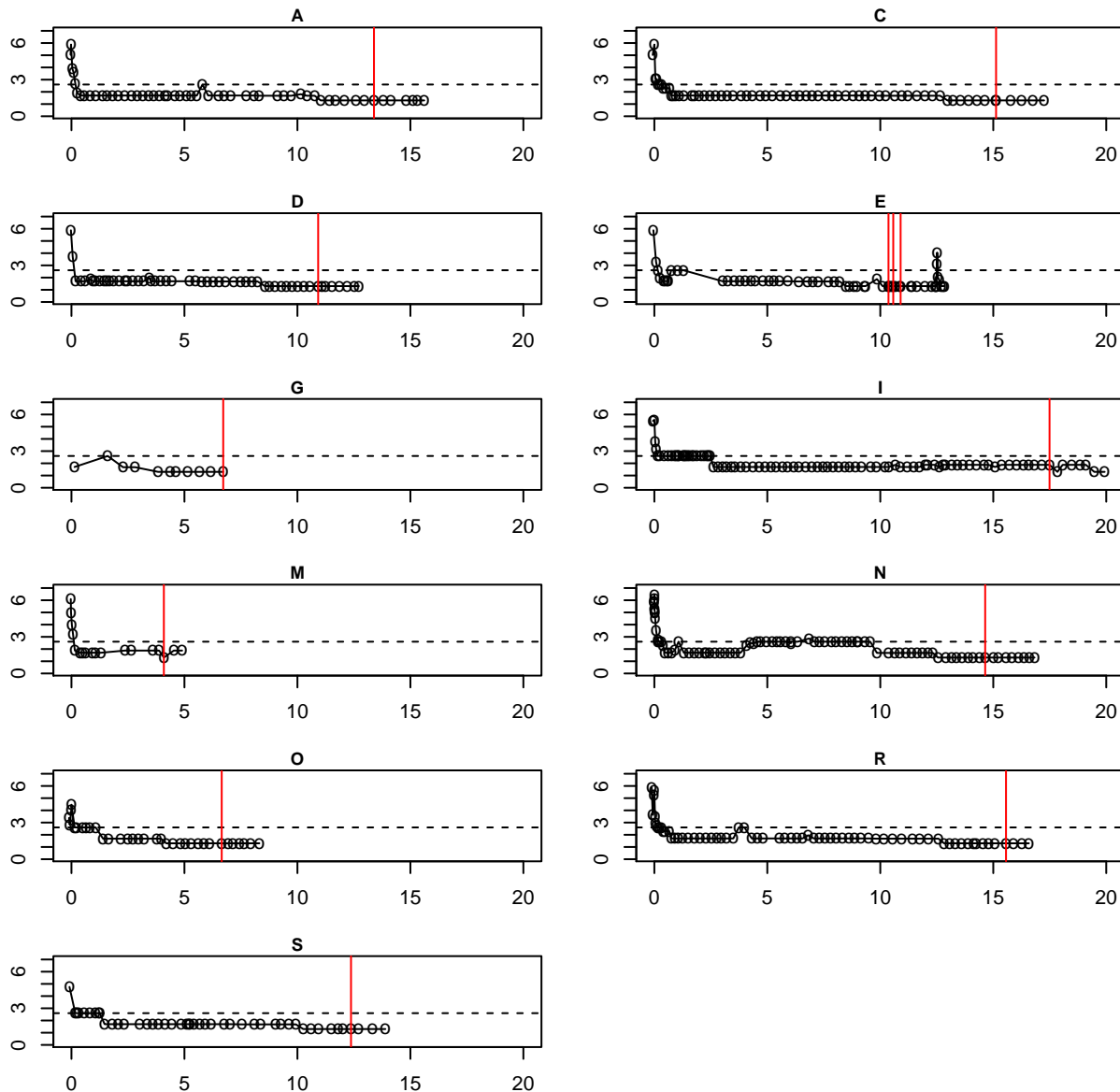

Years since ART initiation

Supplement: S1 Fig — Letter above each plot corresponds to participant identifier listed in Table 1. Black dashed horizontal line indicates 400 c/mL. Red vertical line indicates time of observation for study visit/s in this report. Data preceding zero on the x-axis indicate pre-ART plasma viral load measurements. Viral loads at the limits of detection (e.g., <400, <50, etc.) were set to the limit of detection (e.g., 400, 50, etc., respectively). ART: antiretroviral therapy, c/mL: copies of HIV RNA per milliliter of blood plasma. (PDF) [file pone.0170548.s001.pdf]
